# Making Visible HTLV Infection in a Non-endemic Area of Argentina

**DOI:** 10.3389/fmed.2022.892159

**Published:** 2022-07-08

**Authors:** Federico R. Simioli, Maria B. Bouzas, Dana Mijalovsky, Maria V. Pineda, Lilia Mammana, Andrea Mangano, Tomas A. Orduna

**Affiliations:** ^1^Centro Municipal de Patología Regional Argentina y Medicina Tropical (CEMPRA-MT), Hospital de Infecciosas F.J. Muñiz, Ciudad Autónoma de Buenos Aires, Argentina; ^2^Division Análisis Clínicos, Hospital de Infecciosas F.J. Muñiz, Ciudad Autónoma de Buenos Aires, Argentina; ^3^Unidad de Virología y Epidemiología Molecular-CONICET- Hospital de Pediatría “Prof. Dr. Juan P. Garrahan, ” Ciudad Autónoma de Buenos Aires, Argentina; ^4^Unidad de Virología, División Análisis Clínicos, Hospital de Infecciosas F.J. Muñiz, Ciudad Autónoma de Buenos Aires, Argentina; ^5^Consejo Nacional de Investigaciones Científicas y Técnicas (CONICET), Ciudad Autónoma de Buenos Aires, Argentina

**Keywords:** HTLV-1/2 infection, HTLV outpatient unit, HTLV follow-up, non-endemic area, neglected disease

## Abstract

In Argentina, the human T-cell lymphotropic virus type 1 (HTLV-1) infection has been documented mainly among blood banks with a prevalence of ~0.02–0.046% for Buenos Aires city, 0.8% for the northeast, and 1% for the northwest; both areas are considered endemic for HTLV-2 and 1, respectively. Policies and specific guidelines for testing blood donors for HTLV are included since 2005. Screening for antibodies is performed at blood banks and confirmatory testing is performed at reference laboratories. There are no specific recommendations for the assistance of communities and individuals affected, nor referral to specialized clinics on the HTLV infection. In 2016, as a strategy of intervention, we opened a specialized clinical attendance in a referral infectious diseases public hospital for the comprehensive approach to patients with HTLV, offering follow-up and counseling for patients and their families for the early diagnosis of HTLV-1/2 and related diseases. During the study, 124 patients with presumptive HTLV positive diagnosis from blood bank, symptomatic patients (SPs), relatives, and descendants visited the unit. A total of 46 patients were HTLV positive (38 HTLV-1 and 8 HTLV-2). There were nine SPs (2 adult T-cell leukemia/lymphoma [ATL] and 7 HTLV-1-associated myelopathy/tropical spastic paraparesis [HAM/TSP]). All patients with HTLV-1 and−2 were offered to study their relatives. Two out of 37 (5.4%) descendants tested were positive for HTLV-1. Sexual partners were studied; among 6 out of 11 couples (54.5%) were found positive (5 HTLV-1 and 1 HTLV-2). Other relatives, such as mothers (1/2) and siblings (1/6), were positive for HTLV-1. According to the place of birth among HTLV-1 carriers, 58% were born in an endemic area or in countries where HTLV infection is considered endemic while for HTLV-2 carriers, 12.5% were born in an endemic area of Argentina. The proviral load (pVL) was measured in all, patients with HTLV-1 being higher in symptomatic compared with asymptomatic carriers. In addition, two pregnant women were early diagnosed during their puerperium and breastmilk replacement by formula was indicated. Inhibition of lactation was also indicated. Our study provides tools for a multidisciplinary approach to the infection and reinforces the importance of having specialized clinical units in neglected diseases, such as HTLV for counseling, clinical and laboratory follow-up, and providing useful information for patients for self-care and that of their families.

## Introduction

The human T-cell lymphotropic virus type 1 (HTLV-1) belongs to the Retroviridae family and the Delta retrovirus genus, together with HTLV-2 and bovine leukemia virus (BLV) and the recently discovered HTLV-3 and HTLV-4 (1). The number of HTLV-1-infected people was first estimated between 10 and 20 million (2), and more recently, between 5 and 10 million. Considering that many countries still have undetermined HTLV-1 seroprevalence, some authors consider the latest could be underestimated (3). HTLV-1 is found in highly endemic areas, such as Japan, sub-Saharan Africa, the Caribbean region, and South America. Smaller infection foci have been described in the Middle East, Romania, and Australo-Melanesia (3).

Human T-cell lymphotropic virus type 1 is the etiologic agent of adult T-cell leukemia/lymphoma (ATL) and may lead to the development of chronic progressive myelopathy named HTLV-1-associated myelopathy/tropical spastic paraparesis (HAM/TSP) (4–7). Other inflammatory diseases, such as uveitis, dermatitis, and arthritis, have also been associated demonstrating a broad spectrum of systemic inflammation caused by the viral infection (8–10). The majority of HTLV-1 carriers remain asymptomatic throughout their lives, while 2.5–5% developing ATL and 0.3–2% developing HAM/TSP; the risk of developing the disease is related to the time of infection and the route of transmission (11). In contrast, the etiological role of HTLV-2, despite its initial isolation from two patients with hairy T-cell leukemia and a few cases of HAM-like disease, has not yet been established (12–14). HTLV-1 is primarily transmitted by three main routes: vertical transmission from mother-to-child after prolonged breast-feeding (15), non-protected sexual intercourse (16), and contamination with blood products (cellular components, organ and tissue transplantation, and sharing contaminated needles) (17). The diagnosis of HTLV-1/-2 infection relies on the detection of antibodies and combines at least one or two inmunoassays, mainly enzyme immunoassays (EIAs), followed by a confirmatory test for reactive samples, such as Western blot or line immunoassay (LIA), with the additional information regarding subtype. For indeterminate samples, molecular testing allows for final confirmation and subtyping, and HTLV-1 DNA proviral load (pVL) has been proposed as a biomarker in the disease progression during the clinical follow-up (18).

In Argentina, HTLV-1 infection has been documented mainly among blood banks with a prevalence of 0.02–0.046% for Buenos Aires city, 0.8% for the northeast, and 1% for the northwest of the country. Both areas are considered endemic for HTLV-2 and 1, respectively (19–21). In addition, the northwest is the region with higher incidence for HAM/TSP (22, 23). Although the disease burden due to HTLV-1 is unknown, in our country, cases of ATL are diagnosed annually and cases of infectious dermatitis have been reported (24–26). In people living with HIV, it has been reported a prevalence for HTLV-1/2 between 11 and 19% since 1990, actually a decline has been observed (27–32). As it has been recognized by the WHO's last report, there are gaps in HTLV-1 prevalence in South America and a global lack of systematically collected data on HTLV-1-related diseases (18).

The City of Buenos Aires is a non-endemic area for these viruses and is a cosmopolitan city that receives a large number of immigrants every year from different areas from Argentina and South America, some of them from endemic areas for HTLV-1. Argentina has guidelines for testing blood donors with no specific recommendations for the assistance of communities and individuals affected by HTLV-1 infection. In 2016, as a strategy of intervention, we opened a specialized clinical attendance for the comprehensive approach of HTLV carriers to implement follow-up and counseling for patients and their families for the early diagnosis of HTLV-1/2 related diseases and to provide medical care. This study aimed to describe the results from the implementation, counseling, and follow-up of HTLV carriers attending at an infectious diseases public hospital during the period 2016–2021.

## Materials and Methods

**Figure 1:**
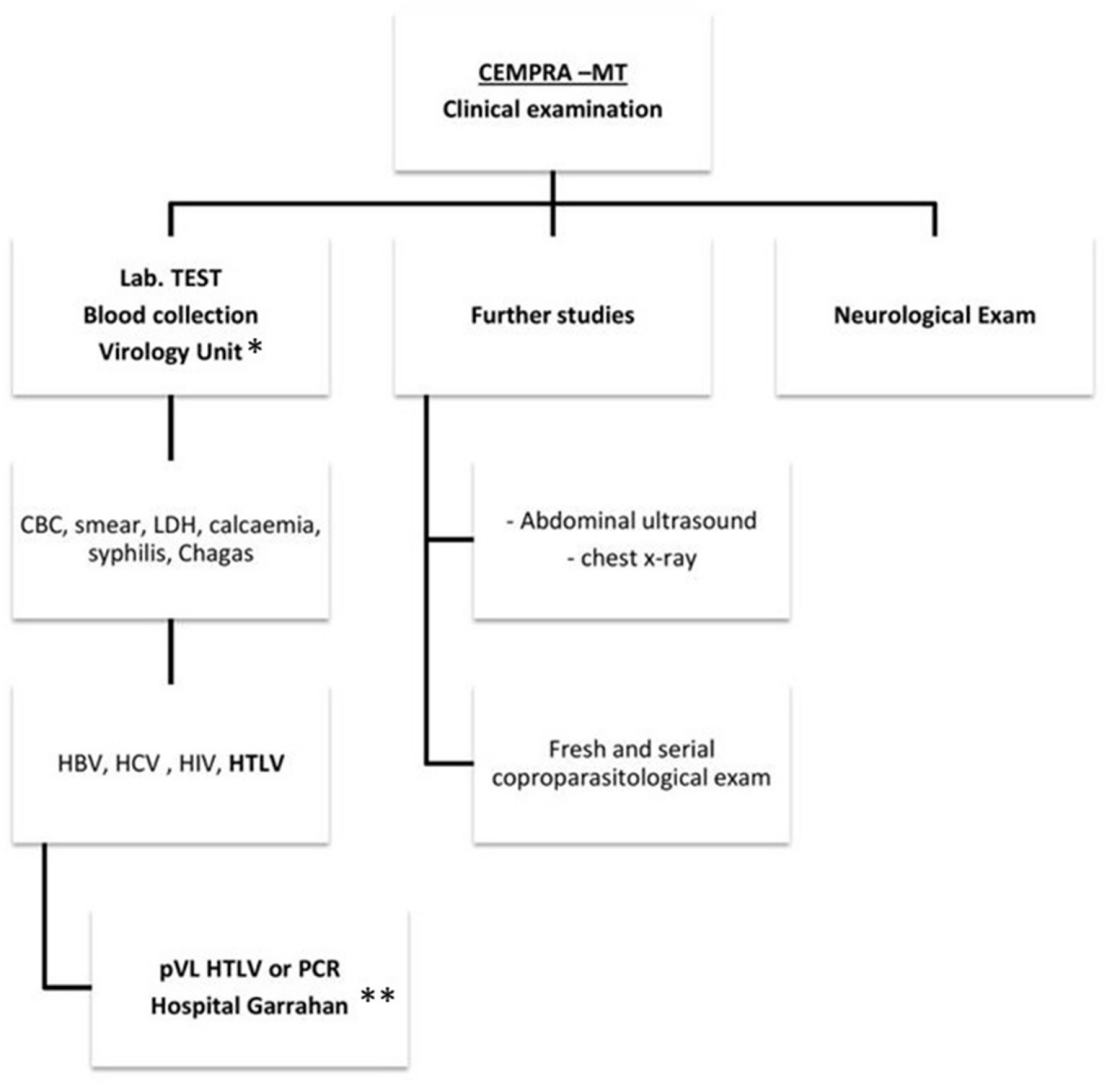
A clinical care flowchart at Centro Municipal de Patología Regional Argentina y Medicina Tropical (CEMPRA-MT). *Unidad de Virología, Hospital Muñiz. **Unidad de Virología y Epidemiologia Molecular, Hospital Garrahan. CBC, cellular blood count; LDH, lactate dehydrogenase.

Clinical attendance for HTLV carriers was implemented in the outpatient Centro Municipal de Patología Regional Argentina y Medicina Tropical (CEMPRA-MT) unit at the Hospital Muñiz, which is a centenary referral infectious disease public hospital from the government of Buenos Aires city. We started in April 2016 and patients were scheduled once a week and two medical doctors were responsible for the attendance. Blood donors were referred from blood banks and HTLV carriers were referred to our clinic from other hospitals. The first visit includes counseling, physical and neurological examination, and a laboratory test (Figure 1). Complementary studies included chest X-ray and abdominal ultrasound. Demographic and epidemiological data (sex, age, place of birth of the patient and their parents, sexual partners and number of descendants and length of lactation, transfusions received, organ transplants, and intravenous drugs usage) was obtained, and information was collected through a structured interview. All these data were recorded on an electronic database. Once patients have confirmed their virological status for HTLV-1/2 infection, antibody testing was offered to sexual partners, siblings, and descendants living in Buenos Aires. Medical consultations were made with the neurology, oncology, or dermatology units as required.

The protocol was approved by the institution review board of the Hospital Garrahan (Number 639). Informed signed consent was obtained from patients.

### Laboratory Studies

Serum samples were studied for HCV antibodies, HBsAg, and Anti HBc for hepatitis B by quimioluminiscence (CMIA, Architect^®^, Abbott) until 2018 and since 2019 by electroquimioluminiscence (ECLIA, Elecsys^®^, Roche, Mannheim). Antibodies for Chagas disease and for syphilis were studied by quimioluminiscence (CMIA, Architect^®^, Abbott) following a reverse algorithm, respectively. In all cases, assays were performed following the manufacture's instructions. For HIV testing, samples were studied by a fourth-generation assay (Duo^®^, Biomerieux), and reactive samples were further confirmed following the national algorithm that includes viral load.

Biochemical markers include cellular blood count (CBC) with smear, evaluated by a hematologist, lactate deshidrogenase (LDH), and calcemia which were performed on a Cobas C501^®^ (Roche). Fresh and serial coproparasitological studies were requested in search of *Strongyloides stercoralis*, including plate culture in nutrient agar.

#### HTLV Studies

**Figure 2:**
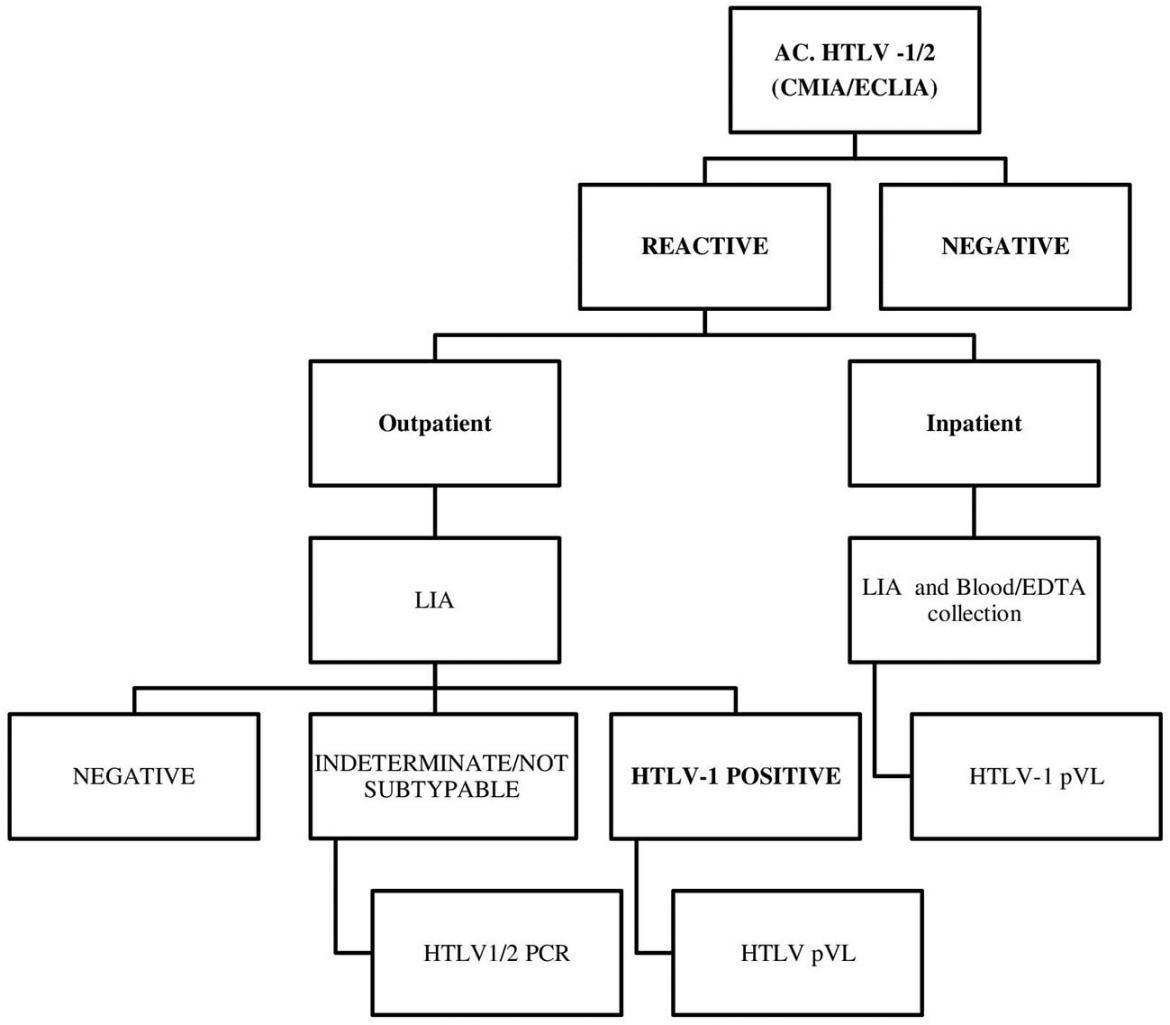
A human T-cell lymphotropic virus (HTLV) diagnostic algorithm.

Blood samples were collected in advance for all HTLV studies to avoid recitation of the patients and following the diagnostic algorithm described (Figure 2). Antibodies for HTLV-1 were screened by quimioluminiscence [rHTLV I/II (CMIA, Architect^®^, Abbott)] until 2018 and since 2019 by electroquimioluminiscence Elecsys^®^ HTLV I/II (Roche, Mannheim) and reactive samples were tested by LIA (INNO-LIA^®^ HTLV I/II Score, Fujirebio, Japan) as a confirmatory test following the manufacturer's instructions. A qualitative PCR was performed for untyped virus results. Confirmed HTLV-1 samples were further tested for pVL for HTLV-1 in peripheral mononuclear cells (PBMCs) by an in-house real-time quantitative PCR (qPCR) as previously described (33). The assay had a detection limit of 400 HTLV-1 copies/10^6^ PBMCs, with a broad range of quantitation (2.6 log_10_ to −6 log_10_) and a total error accepted of 0.5 log_10_. Both molecular studies were performed at the Virology Unit in the Hospital Garrahan.

### Follow-Up

Subsequent visits were planned individually: for asymptomatic carriers (ACs), annual clinical and laboratory controls, such as pVL, were assigned, and for symptomatic patients (SPs), visits were scheduled according to the needs for each patient, such as consultations, laboratory testing, and referral, to other centers if required. For ACs, they were informed to acquire an annual control through a phone number destined for it. For patients who did not assist to their medical visit, they received a call back to promote it.

### Statistical Analysis

Standard descriptive statistics were used to describe the baseline characteristics of the population. Results were expressed as median and interquartile range (IQR). The Mann–Whitney *U*-test was used to compare continuous data between ACs and SPs. A two-sided *p* < 0.05 was considered to indicate statistical significance. Data were analyzed using the Graphpad Prism 5 software package 4.91.

## Results

During the studied period (April 2016–March 2021), a total of 124 individuals visited our outpatient unit, including those with presumptive HTLV positive diagnosis from blood bank (*n* = 56), SPs (*n* = 12), relatives (*n* = 19), and descendants (*n* = 37). Of them, 46 were enrolled for the follow up study with confirmed HTLV positive diagnosis. Furthermore, 32 patients with presumptive HTLV diagnosis were found to be negative for HTLV antibodies, 29 at the time they were retested (false positive from blood banks), and three patients with a presumptive diagnosis of HAM/TSP. Molecular studies were not performed on these three individuals due to the final diagnosis, which included severe discopathy in one case, and peripheral neuropathy in the other two. Table 1 describes the relationship between index cases of HTLV-1 and−2 and their family members.

**Table 1:** Relationship between index cases of human T-cell lymphotropic virus (HTLV)-1 and−2 and family members.

**Ctable 1ase *N*°**	**Index cases of HTLV-1**	**Sex**	**Sexual partner**	**Mother**	**Sibling**	**Descendent**
1	ATL	M	**Negative**	Not tested	**1 positive HTLV-1 (F) and 5 negative**	Without
2	ATL	F	Without	Not tested	Not tested	**1 negative**
3	Blood donor	F	Without	Without	Without	Without
4	Blood donor	F	**positive HTLV-1 (M)**	Not tested	Not tested	**3 negative**
5	Blood donor	F	Not tested	Not tested	Not tested	3 not tested
6	Blood donor	F	Not tested	Not tested	Not tested	**1 negative and 1 not tested**
7	Blood donor	F	Not tested	Not tested	Not tested	2 not tested
8	Blood donor	M	**Negative**	Not tested	Not tested	**3 negative**
9	Blood donor	M	Not tested	Not tested	Not tested	2 not tested
10	Blood donor	F	Not tested	Not tested	Not tested	2 not tested
11	Blood donor	M	Without	Not tested	Not tested	**4 negative**
12	Blood donor	F	**Positive HTLV-1 (M)**	Not tested	Not tested	**3 negative**
13	Blood donor	F	Not tested	Not tested	Not tested	**1 negative**
14	Blood donor	F	Without	Not tested	Not tested	Without
15	Blood donor	F	Without	Not tested	Not tested	2 not tested
16	Blood donor	F	Not tested	**Positive HTLV-1 (F)**	Not tested	2 not tested
17	Blood donor	F	**Negative**	Not tested	Not tested	**1 positive HTLV-1 (F) and 2 not tested**
18	Blood donor	F	**Negative**	**Negative**	Not tested	Without
19	Blood donor	F	Without	Not tested	Not tested	**3 negative**
20	Blood donor	M	Without	Not tested	Not tested	1 not tested
21	Blood donor	F	Without	Not tested	Not tested	**2 negative**
22	Blood donor	M	Without	Not tested	Not tested	Without
23	HAM/ TSP	F	Not tested	Without	Not tested	Without
24	HAM/ TSP	F	**Positive HTLV-1 (M)**	Without	Not tested	2 not tested
25	HAM/ TSP	F	**Positive HTLV-1 (M)**	Without	Not tested	**1 positive HTLV-1 (M), 1 negative and 1 not tested**
26	HAM/ TSP	M	Not tested	Not tested	Not tested	Without
27	HAM/ TSP	F	**Positive HTLV-1 (M)**	Not tested	Not tested	2 not tested
28	HAM/ TSP	M	Without	Not tested	Not tested	**3 negative**
29	HAM/ TSP	M	Without	Not tested	Not tested	Without
	**Index cases of HTLV-2**	**Sex**	**Sexual partner**	**Mother**	**Sibiling**	**Descendent**
30	Blood donor	F	**Negative**	Not tested	Not tested	**3 negative**
31	Blood donor	F	**Positive HTLV-2 (M)**	Not tested	Not tested	**3 negative**
32	Blood donor	F	Not tested	Not tested	Not tested	**4 negative**
33	Blood donor	F	Not tested	Not tested	Not tested	Without
34	Blood donor	M	Not tested	Not tested	Not tested	2 not tested
35	Blood donor	F	Not tested	Not tested	Not tested	1 not tested
36	Blood donor	F	Without	Not tested	Not tested	Without

*M, male; F, female. Bold type for family members studied*.

All 46 HTLV carriers (38 HTLV-1 and 8 HTLV-2) were clinically evaluated. Among the HTLV-1 group, 76% were asymptomatic carriers (29/38) and the source of identification was: 20 blood donors, 5 sexual partners, 1 sibling, 1 mother, and 2 descendants. Among SPs, 2 patients were diagnosed as ATL and 7 patients had HAM/TSP. According to the age at enrollment for HTLV-1, SP and AC had a similar median age of 46 years (IQR = 31–52) and 44 years (IQR = 40–51), respectively. For HTLV-2 carriers, the median age at enrollment was 46 (IQR = 40–52). According to the place of birth among HTLV-1 carriers, 58% were born in an endemic area of Argentina or in other countries where HTLV infection is considered endemic. Moreover, when we analyzed AC and SP, we found that 59 and 56% for the respective groups were born in endemic areas. For HTLV-2 infection, 12.5% of them were born in an endemic area of Argentina. Table 2 shows a summary of HTLV-1 and HTLV-2 carriers during follow-up.

**Table 2:** Summary of the demographic and clinical characteristics of the HTLV-1 and −2 cases observed during follow-up.

	**HTLV-1 Cases**		**HTLV-2 Cases**
	**(** ***N*** **:38/46)**		**(*N*: 8/46)**
	AC	SP	AC
	29/38 (76.3%)	9/38 (23.7%)	8/8 (100%)
Age (median)	44 years (IQR = 40–51)	46 years (IQR = 31–52)	46 (IQR = 40–52)
Sex	18/29 (62%) women	5/9 (55.5%) women	6/8 (75%) women
Birthplace	3 endemic area of Argentina (Salta, Santiago del Estero)	3 non-endemic area of Argentina.	1 endemic area of Argentina (Chaco)
	17 non-endemic area of Argenttina		7 non-endemic area of Argentina
	9 endemic area outside Argentina (Paraguay, Peru, Bolivia and Rep. Dominicana)	6 endemic area outside Argentina (Paraguay, Peru, Bolivia and Rep. Dominicana)	
Coinfection	1 Chagas disease	2 HAM/TSP coinfected with HIV, HBV and HCV	1 HIV
		1 HAM/TSP coinfected with TB and syphilis	
		1 HAM/TSP with Chagas disease	
HTLV-1 pVL (median)	4.75 log_10_ HTLV-1 copies /10^6^PBMCs	5.25 log_10_ HTLV-1 copies /10^6^PBMCs	Not Aplicable
	(IQR 4.08-5.07)	(IQR: 4.65-5.82)	

*AC, asymptomatic carriers; SP, symptomatic carriers; HTLV-1 pVL, HTLV-1 proviral load; TB, tuberculosis; HIV, human immunodeficiency virus; HCV, heaptitis C virus; HBV, hepatitis B virus; PBMC, peripheral mononuclear cells; IQR, interquartile range*.

Serology for hepatitis B virus (HBV), hepatitis C virus (HCV), and HIV were found positive in 2 HTLV-1 SP (2/46: 4.3%), and Chagas disease in 1 HTLV-1 SP and 1 AC (2/46: 4.3%). Coinfection with *tuberculosis* (TB) was found in one HAM/TSP patient also with a diagnosis of syphilis (1/46: 2.2%; Table 2).

Specimens for coproparasitological studies were available in 17/46 patients, all being negative for *S. stercoralis*. Among HTLV-2 carriers' coinfection with HIV was documented in one case (Table 2).

All HTLV-1 and−2 carriers were offered to study their relatives. The overall number of descendants were 60, with 37 (62%) were available and willing to be tested, 2 of whom were HTLV-1 positive. The remaining descendants reside outside the Buenos Aires city. Sexual partners were studied; among 6/11 couples (54.5%) were found positive (5 HTLV-1 and 1 HTLV-2). Other relatives, such as mothers (*N*: 2) and siblings (*N*: 6), were tested, being 1 mother and 1 sibling positive for HTLV-1 as is shown in Table 2.

The increase in HTLV carriers enrolled in the years between 2016 and 2019 was 1.8, 1.9, and 0.8, respectively (2016: *N*: 5 patients; 2017: *N*: 9 patients; 2018: *N*: 15 patients; 2019: *N*: 12 patients; 2020: *N*: 4 patients; and 2021: *N*: 1 patient). All SPs and ACs returned for medical controls, and the average number of visits was 7 (2–13) and 3 (2–7), respectively during the study period 2016–2021. It is noteworthy that 52% of HTLV-1 ACs had more than 2 clinical visits and 45% had more than two HTLV-1 pVL.

**Figure 3:**
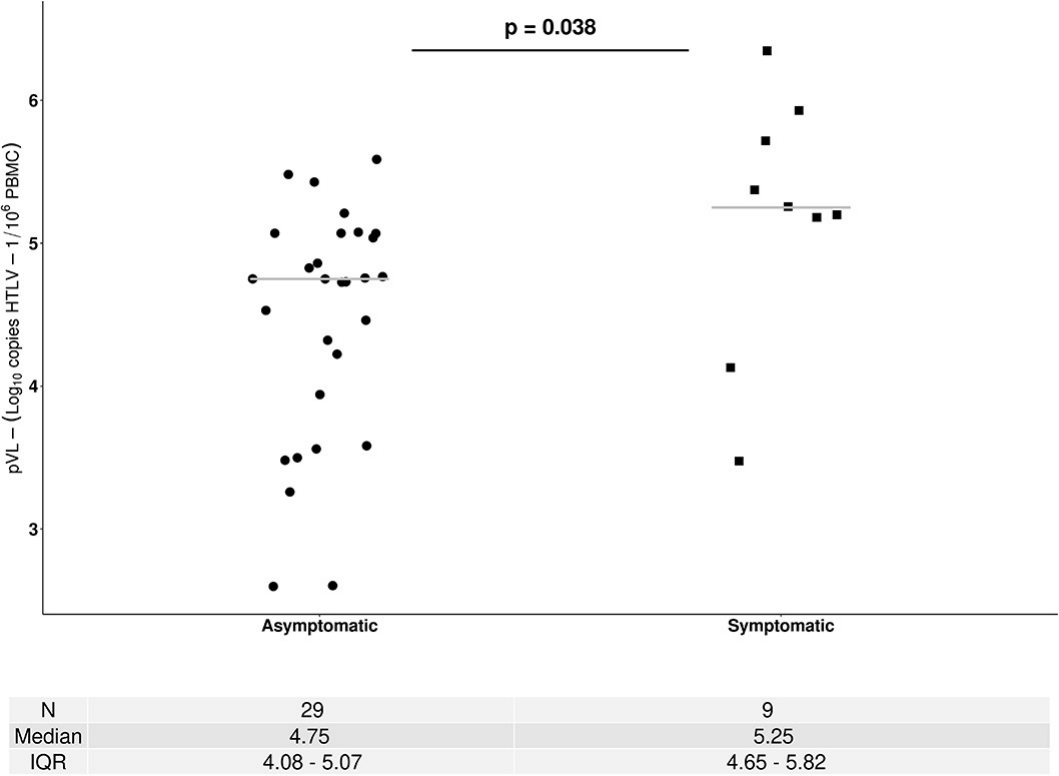
Human T-cell lymphotropic virus 1 proviral load (pVL) among asymptomatic carriers (ACs) and symptomatic patients (SPs). HTLV-1 pVL values correspond to samples collected at first visit. • AC, ▴ ATL, ■HAM/TSP. The Mann–Whitney *U*-test was used to compare continuous data between ACs and SPs. A two-sided *p* < 0.05 was considered to indicate statistical significance.

A total of 72 pVLs corresponding to 38 HTLV-1 carriers were done, and 19 of them (14 ACs and 5 SPs) had more than one pVL measure. The median pVL HTLV-1 at baseline (first clinical visit) corresponding to 29 ACs and 9 SPs was 5.25 log_10_ HTLV-1 copies/10^6^ PBMCs among SPs and 4.76 log_10_ HTLV-1 copies/10^6^ PBMCs ACs (*p*: 0.038) as is shown in Figure 3. The average of the difference among pVL measures in AC was 0.28 log_10_ (data not shown) which in all cases was inferior to the total error of the assay.

Breastmilk replacement by formula feeding and inhibition of lactation was started in 2 women that were breastfeeding at the time of HTLV-1 diagnosis. Both women were derived from the public maternity of Buenos Aires during their puerperium with previous positive results from blood bank. The two babies born tested negative, one of them at 3 and 12 months of age, and the other one at 24 months of age.

During the studied period, none of the HTLV-1 AC evolved to a symptomatic stage.

Additionally, two of the ATL cases underwent treatment with CHOEP chemotherapy, AZT/3TC, and alpha interferon and were derived to be treated in specialized onco-hematology centers. In one of them, a chemotherapy scheme of 6 cycles was prescribed, the length of follow-up was 3 years, and pVL was performed on average every 3 months during the first 2 years. Once treatment was completed (average duration: 6 months) a decline of 0.7 log_10_ was observed (pVL_1_: 6 log_10_; pVL_3_: 5.3 log_10_). Subsequently, pVLs remained stable for an average period of 30 months (mean pVL: 5.34 log _10_).

Treatment among patients with HAM/TSP included, in 5 of them, corticosteroid therapy, symptomatic treatment, and physiatry.

Only one HTLV-1 carrier died, she was a patient with HAM/TSP and the cause of death was a colon cancer.

## Discussion

Human T-cell lymphotropic virus type 1 is associated with high morbidity and mortality clinical conditions, while HTLV-2 is rarely associated with disease. Despite the worldwide distribution, the infection appears to be geographically concentrated and, except from Japan, low- and middle-income countries and ethnic minority population groups in high-income nations are the most affected (2, 3). The majority of infected carriers will remain asymptomatic throughout their entire life, but many of them will develop one or more of a range of diseases. At this point, ATL is an aggressive type of leukemia/lymphoma characterized by a short survival time and a poor response to chemotherapy, and HAM/TSP produces a disability that greatly impacts patient's autonomy and quality of life, up to 50% becoming wheelchair dependent.

Argentina has an endemic area for HTLV-1 in the northwest (around Jujuy province), which is reflected in the prevalence among blood banks, pregnant women, and the high incidence of TSP/HAM (29). The northeast (around Formosa and Chaco provinces) is also considered to be an endemic focus, mainly for HTLV-2, as it has been reported in terms of the prevalence among Amerindian communities and the highest prevalence described in blood banks (23, 33–36).

Buenos Aires city is a non-endemic area, with a prevalence among blood donors between 0.02 and 0.046% similar to other provinces in the center of the country (20). The frequency of positivity for HTLV-1 has been higher than HTLV-2 from the first reports up to the present (19, 21). The difference observed between HTLV-1 and−2 cases in endemic areas could reflect the higher prevalence of HTLV-1 infection compared with HTLV-2 in Argentina. On the other hand, HTLV-2 infection is mainly restricted to a small focus among Amerindian population in the northeast of our country. Even during the 1990, the prevalence for HTLV-1 among people living with HIV was higher to equal than HTLV-2 in contrast to what has been reported in the United States and Europe.

The outpatient unit described here was implemented to offer medical care and counseling to individuals with previous results from blood banks and SPs referred to our hospital (public infectious disease hospital). We found a high proportion of HTLV-1 carriers (38/46), being the source of identification blood donors in 56/124 (45%). It is important to highlight that 52% (29/56) of them were negative at that time, were retested. Considering the sensitivity and specificity of screening assays, this data reinforces the need for confirmation of blood donors' results and the respective counseling to minimize the psychological impact for patients.

Clinically, as expected all HTLV-2 carriers were asymptomatic being mainly (87.5%) born in non-endemic areas. None of them were intravenous drug users, one was born in an endemic area for this virus (Chaco province), the second had a coinfection with HIV, and the only risk factor was considered to be the sexual transmission. Three carriers received blood transfusions in 1989, 1996, and 2015, since blood bank screening started in Argentina in 2005, two of them could acquire the infection through that route. The latest two cases were from the areas where HTLV infection is limited, one from Corrientes province and the other one from Santiago del Estero. Remesar et al. (20) described no statistic association between HTLV-2 infection and birth of place among blood donors.

Among HTLV-1 carriers, 76.3% were asymptomatic during their follow-up and despite their clinical condition, 59% of them were from endemic areas, in contrast to HTLV-2. This is important to highlight that Remesar et al. found an overall seroprevalence of 0.046% for HTLV-1/2 at the Hospital Garrahan Blood bank, an association between HTLV-1 prevalence and North Argentina as the blood donor birth place was found, unlike HTLV-2. The authors found a relatively high prevalence for HTLV-1 (0.19%) among blood donors from non-endemic areas (20). This has been reported by Biglione et al. (21) that 30% (0.01% prevalence) of HTLV positive blood donors were born in HTLV-1/2 South American endemic areas. It seems that the percentage of HTLV carriers with no relation to endemic areas has increased from initial reports (20, 36).

Moreover, the prevalence among blood banks at the Blood Bank National Program during 2019 (total number of screenings: 799.388) was 0.22% for HTLV-1/2 (Dr. Pisarello personal communication). For the public health system in Buenos Aires city, during the year of 2021 (total number of screenings: 43.447), the prevalence rate for HTLV-1/2 was 0.16% of which more than 70% of the samples were confirmed (Dr. Torres personal communication). Even though Buenos Aires city has a regional blood center, this only centralizes all nucleic acid testing from public health institutions, and HTLV is not included in the molecular panel. Screening for HTLV is performed in each blood bank by antibody detection using ELISA and particle agglutination technique. Confirmatory testing for HTLV is not usually performed in blood banks in Buenos Aires. Reactive samples are referred to a few centers that performed confirmatory testing, and the blood bank returns the result to the donor.

Collectively, as a consequence of migration, more HTLV carriers have moved from endemic to non-endemic areas, contributing silently to the transmission of these viruses. This has been also described by Chihara et al., describing the increase in the incidence of ATL in non-endemic areas in Japan and the United States (37).

The pVL was measured in all HTLV-1 carriers. In concordance with previous results, we found that SP had higher values than asymptomatic patients and this was statistically significant (33). No asymptomatic patients evolved to a symptomatic stage, and the difference between pVL values in those with two or more measures was inferior to the total error of the assay.

Coinfection between HTLV-1/2 and HIV has been extensively reported. In our country, since 1990, the prevalence rate in people living with HIV (PLWH) have been reported to be between 11 and 19%. In 2015, we found a decline in the prevalence rate of HTLV-1/2 among PLWH to 5% and among those HTLV/HIV coinfected, 40% were also coinfected with HBV and HCV. In the present study, coinfection with HBV, HIV, and HCV was documented among two patients with HAM/TSP (4.2%) (32). As it has been reported for HCV, the difference in the prevalence rate of coinfection HIV, HCV, and HTLV could be related to the decrease in injection drugs users (IDU) in the study population since this is a very efficient route of infection transmission. The prevalence rate for HCV has dropped in most countries, with the exception of those where IDU is the main source of new infections (38).

It has been suggested that TB could influence in the development of HAM/TSP (39). We found coinfection in one HAM/TSP case, although no studies in Argentina have search the association of TB and HTLV-1 in endemic areas.

Infection with *S. stercoralis* may influence HTLV-1-related disease progression because screening and treatment are performed in centers caring of people with HTLV-1. Although this association has not been reported in Argentina, we include the screening for our patients and we were not able to study all for *S. stercoralis* because of the complexity in sample collection. Screening techniques using urine represent a diagnosis opportunity to consider and implement because it is a sample easy to collect by patients. (40).

All HTLV-1 and−2 carriers were offered to study their relatives, and we found among them that 54.5% of the sexual partners tested positive. Furthermore, the percentage of positive family members (sexual partners, descendants, and others) over the total number of HTLV cases in our group amounts to 21.7% (10/46). This observation reinforces the importance of offering the study to family members. During medical care, it is also important to emphasize the use of condoms in serodiscordant couples or in patients without a stable partner, and avoid exposure to fluids with biological risk, such as blood.

Vertical transmission of HTLV occurs primarily through breastfeeding and not transplacentally or during delivery (41). The risk of transmission increases with longer breastfeeding and high maternal pVL. The seroprevalence of HTLV-1 among pregnant women ranges from 400 to 500 for 10,000 in highly endemic areas of Japan, whereas in non-endemic areas of this country it varies from 10 to 100 for 10,000 (41). For European countries, the seroprevalence is ~4.4 for 10,000. In Argentina, only one study has been conducted in Cordoba province, and Trenchi et al. (42) found a seropositivity of 19 for 10,000. A retrospective study in one center in Jujuy province found a prevalence ranged 60–140 per 10,000 (unpublished data). In our study, 2 women were confirmed as HTLV-1 positive during puerperium, breastfeeding was replaced by formula fed provided through the Argentinean National maternal child program. This implementation was successful since the two babies born tested negative after 1 and 2 years, respectively. As has been suggested, studies among pregnant women may probably reflect the prevalence rates of the general population better than blood donors (41). This reinforces the need to include HTLV in prenatal screening as one of the best strategy to avoid mother-to-child transmission of HTLV-1/2. Furthermore, even in non-endemic areas, prenatal screening is cost-benefit (18).

Since the beginning of the study, the number of HTLV carriers enrolled in the follow-up increased. A reduction in care for other diseases was observed for the period 2020–2021 because of the impact of the COVID-19 pandemic. Beside this, we considered that adherence to follow-up in terms of visits average and pVL performed for SP and AC is remarkable. To facilitate this, we coordinate clinical and laboratory visits on the same day with a comprehensive approach through case management.

Collectively, this implementation shows that there is an unattended need for medical care of HTLV carriers in the health system. It is necessary to design operational care processes linking blood banks with specialized care centers. This will help avoid lost patients and missed opportunities in diagnosis and counseling. These findings also highlight the need for HTLV screening during pregnancy. On the other hand, our results raise the question whether these clinics reflect the situation of the community in terms of the epidemiology of HTLV-1/2 infection and related diseases.

Our study provides tools for a multidisciplinary approach of the infection, and reinforces the importance of having specialized clinical units in neglected diseases, such as HTLV for counseling, clinical and laboratory follow-up, and providing useful information for patients for self-care and that of their families. Furthermore, these clinics will contribute to decrease the misdiagnosis of these infections mainly in non-endemic areas and systematically collect data on the HTLV-1-related diseases leading to a national registry with mandatory reporting.

## Data Availability Statement

The raw data supporting the conclusions of this article will be made available by the authors, without undue reservation.

## Ethics Statement

The studies involving human participants were reviewed and approved by Hospital de Pediatría Juan P. Garrahan, Comite Revisor de Proyectos de Investigación y Etica. The patients/participants provided their written informed consent to participate in this study.

## Author Contributions

FRS, conceptualization, investigation, methodology, data curation, writing–original draft, and formal analysis. MBB: conceptualization, data curation, formal analysis, investigation, methodology, writing—original draft, writing—review and editing, supervision, and project administration. DM: conceptualization, investigation, methodology, data curation, and writing–original draft. MVP: conceptualization, investigation, methodology, data curation, writing–original draft, formal analysis, and writing—review and editing. LM: formal analysis, methodology, and writing—review and editing. AM: conceptualization, formal analysis, investigation, methodology, writing—review and editing, supervision, project administration, and funding acquisition. TAO: writing—review and editing, supervision, and project administration. All authors contributed to the article and approved the submitted version.

## Funding

All laboratory and complementary studies were supported by the Hospital de Infecciosas F.J. Muñiz (Ministry of Health, Buenos Aires City Government). Molecular studies of HTLV-1/2 were supported by Grant N° 2010-0502 (PICT CONICET). The authors would like to acknowledge AIDS Healthcare Foundation Filial Argentina for supporting the publication fee of this paper.

## Conflict of Interest

The authors declare that the research was conducted in the absence of any commercial or financial relationships that could be construed as a potential conflict of interest.

## Publisher's Note

All claims expressed in this article are solely those of the authors and do not necessarily represent those of their affiliated organizations, or those of the publisher, the editors and the reviewers. Any product that may be evaluated in this article, or claim that may be made by its manufacturer, is not guaranteed or endorsed by the publisher.
